# Financial decision-making in a community sample of adults with and without current symptoms of ADHD

**DOI:** 10.1371/journal.pone.0239343

**Published:** 2020-10-12

**Authors:** Dorien F. Bangma, Lara Tucha, Anselm B. M. Fuermaier, Oliver Tucha, Janneke Koerts

**Affiliations:** 1 Department of Clinical and Developmental Neuropsychology, University of Groningen, Groningen, The Netherlands; 2 Department of Psychiatry and Psychotherapy, University Medical Center Rostock, Rostock, Germany; Aalborg University, DENMARK

## Abstract

Research found that adults with attention deficit hyperactivity disorder (ADHD) have more problems with financial decision-making than healthy controls. The present study investigates the impact of symptoms of ADHD on impulsive buying and the use of financial decision styles. Furthermore, the influence of personality, symptoms of depression and demographics on the association between ADHD and these aspects of financial decision-making is evaluated. A community sample of 1292 participants (age range 18–93 years, 45.4% male) completed questionnaires related to ADHD, impulsive buying, financial decision styles, personal financial situation, depression and personality. Four groups were formed based on self-reported ADHD symptoms: an ‘ADHD’ group (n = 45), an ‘Adult-only ADHD’ group (n = 57), a ‘Subthreshold ADHD’ group (n = 162) and a ‘No ADHD’ group (n = 265). Groups were compared using ANOVA and chi-square tests. Furthermore, multiple regression analyses in the complete sample were employed to examine the association between ADHD and financial decision-making. The ADHD and Adult-only ADHD groups reported significantly more impulsive buying, used more often an avoidant or spontaneous decision style and less often saved money compared to the No ADHD group. Regression analyses revealed that impulsive buying and financial decision styles were not significantly associated with ADHD symptoms when controlling for personality, symptoms of depression and demographics. The present study confirms previous research on adults with ADHD by indicating more impulsive buying and a more frequent use of disadvantageous financial decision styles (i.e., avoidant and spontaneous styles) in individuals with an elevated number of current symptoms of ADHD compared to individuals without symptoms of ADHD. Personality and demographic variables were found to be related to both impulsive buying and the use of specific financial decision styles and might be of influence on the association between impulsive buying, the use of financial decision styles and ADHD.

## Introduction

Attention Deficit Hyperactivity Disorder (ADHD), characterized by symptoms of inattention, hyperactivity and impulsivity [1], is historically known as a developmental disorder that only affects children. However, in on average 50% of children with ADHD (32.8%– 84.1% cross-national variation [2]), the condition persists into adulthood resulting in an estimated prevalence of adult ADHD of 3.4% internationally (range 1.2–7.3% [3]). Adults with ADHD have more difficulties in daily life compared to adults without ADHD with, for example, educational and occupational performances and social functioning [4–6]. Adults with ADHD are, furthermore, often involved in risky behavior, such as risky driving, risky sexual behavior or substance abuse [7–10].

Although financial decision-making has hardly been investigated in adults with ADHD, there are indications that adults with ADHD also have problems with this important capability in everyday life. Financial decision-making describes several components related to the ability to manage or direct to manage financial affairs. It involves practical skills and abilities, e.g., counting coins, but also financial knowledge and judgment to perform financial actions [11]. The limited research available indicates an association between symptoms of hyperactivity-impulsivity and inattention and problems with financial decision-making [12–14]. Adults with ADHD are more often financially dependent [12] and report more financial problems compared to age-matched controls (e.g., debts, exceeding credit card limits, difficulties saving money and impulsive buying [4]). A recent study, using standardized objective measures in addition to self-report questionnaires, confirmed that adults with ADHD compared to healthy controls have difficulties with several aspects of financial decision-making, including problems with understanding bank protocols and evaluating financial problems, having debts, not saving money and problems with financial decisions with implications for the future. Furthermore, compared to healthy controls, adults with ADHD were found to have a stronger tendency to buy on impulse and more often used inadequate financial decision styles (i.e., avoiding decisions and spontaneously making a decision) [15].

In the dual pathway model of decision-making, affective processing of information is described as one of two systems involved in decision-making [16–18]. Together with deliberative/analytic processing of information, which strongly relies on cognitive control, both systems are considered to be crucial for adequate decision-making [17,18]. Motivational/affective dysregulation in adults with ADHD [19,20] might, therefore, underlie problems with financial decision-making in adults with ADHD. On the other hand, cognitive dysregulation of adults with ADHD (e.g., impairments in executive functions and numeracy [15,21–23]) might also have an impact on (financial) decision-making [15,24].

Inadequate financial decision-making can have major negative consequences for individuals, such as debts and poverty, and further investigation of this topic in adults with ADHD is, therefore, important. According to the framework of Appelbaum et al. [11], financial decision-making requires adequate financial competence (i.e., practical financial skills, knowledge and the ability to judge and make decisions), but also relies on financial performances. The latter describes the abilities and behaviors that affect the degree of success when making financial decisions, such as the use of specific decision styles or buying on impulse. For example, one may be financially competent but unable to make adequate financial decisions due to deficiencies in financial performance. Research should, therefore, not only focus on financial competence but also on financial performances when studying financial decision-making. Furthermore, according to the framework of Appelbaum et al. [11], contextual factors might also be of influence on both financial competence and financial performances and need to be taken into account.

The aims of the present study are, therefore, (1) to investigate the impact of current symptoms of ADHD on two types of financial performance, i.e., impulsive buying and the use of financial decision styles, (2) to partly replicate a recent study on financial decision-making in adults with ADHD [15] and (3) to determine whether symptoms of depression and personality traits are of influence on the association between impulsive buying, the use of financial decision styles and symptoms of ADHD. The latter are considered to be potentially influential contextual factors and are of interest as symptoms of depression are prevalent in adults with ADHD [25] and because specific personality profiles have been linked to ADHD (see Gomez and Corr [26] for a meta-analytic review). Moreover, personality and symptoms of depression, on itself, have both been discussed in the context of decision-making [27–31]. A community sample is used in the present study which allows a detailed exploration of the associations between impulsive buying, the use of financial decision styles and symptoms of ADHD since not only adults with and without ADHD can be compared, but also comparisons can be made with adults with signs of ADHD that are considered subthreshold for a clinical diagnosis but which still might be of influence on the use of financial decision styles and impulsive buying.

Based on previous research [15], a positive relation is expected between the severity of symptoms of ADHD and the tendency to buy on impulse and the use of inadequate financial decision styles (i.e., an avoidant and a spontaneous decision style [32]). Furthermore, it is expected that individuals who meet self-reported symptom criteria of adult ADHD show more problems with these types of financial performance and in their personal financial situation compared to individuals who do not meet a clinically relevant number of criteria of ADHD [1].

## Materials and methods

### Participants

A community sample of 1292 participants (586 males and 706 females) with an age range of 18 to 93 years (M = 48.8, SD = 18.5) participated in the present study. Demographical characteristics of the participants are described in Table 1. The (para)medical history of participants, beside medication use, has not been recorded. Fifty-six participants (4.3%) reported to use antidepressants or antipsychotics, twenty-one participants (1.6%) reported to use benzodiazepines and four participants (0.3%) reported to use stimulants (e.g., methylphenidate). The reason for medication use was, however, not evaluated and, therefore, participants were not excluded on basis of their medication use.

**Table 1:** Demographic characteristics of total sample and ADHD groups.

	Total sample	ADHD	Adult-only ADHD	Subthreshold ADHD	No ADHD	Group differences		
						*Statistics*	*p*	*d*
**N**	1292	46	57	162	265	-		
**Age *M (SD)***	48.8 (18.5)	31.5 (12.5)	36.7 (16.9)	38.1 (17.2)	37.1 (16.2)	F(3,529) = 2.0	.110	0.213
**Age *range***	18–93	18–69	18–84	18–90	18–93	-		
**Sex *% male / % female***	45.4/54.6	60.9/39.1	40.4/59.6	45.7/54.3	45.7/54.3	*X*^*2*^(3) = 4.8	.187	0.191
**Level of education**^1^^,^^2^	-	-	-	-	-	*X*^*2*^(6) = 3.1	.792	0.155
Lower *%*	9.1	4.3	10.5	6.2	6.5	*X*^*2*^(3) = 1.9	.604	0.118
Intermediate *%*	27.9	30.4	19.3	24.7	24.3	*X*^*2*^(3) = 1.7	.633	0.114
Higher *%*	63.0	65.2	70.2	69.1	69.2	*X*^*2*^(3) = 0.4	.950	0.052
**Employment status**	-	-	-	-	-	*X*^*2*^(15) = 26.4	.035*	0.458
Student *%*	14.7	30.4	40.4	33.3	24.9	*X*^*2*^(3) = 7.1	.070	0.232
Employed (part-time) *%*	20.8	17.4	10.5^a^	17.3	24.9	*X*^*2*^(3) = 8.0	.047*	0.248
Employed (full-time) *%*	32.2	34.8	26.3	30.2	36.6	*X*^*2*^(3) = 3.3	.351	0.159
Unemployed *%*	4.7	6.5	12.3^a^	6.2	3.0	*X*^*2*^(3) = 8.7	.034*	0.258
In retirement *%*	23.1	2.2	7.0	7.4	7.2	*X*^*2*^(3) = 1.7	.635	0.114
Other *%*	4.5	8.7	3.5	5.6	3.4	*X*^*2*^(3) = 3.2	.367	0.155

*Note*. ADHD = Attention Deficit Hyperactivity Disorder; ARS = Attention Deficit Hyperactivity Disorder rating scale.

^1^ Levels of education are based on the Verhage coding system [33] which ranges from 1 (primary school not finished) to 7 (university degree). Verhage classification 1–4 are equivalent to lower education, 5 is equivalent to intermediate education and 6 and 7 are equivalent to higher education.

^2^ Missing data: n = 4 in Total sample and n = 2 in No ADHD group.

* *p* < .05. Post-hoc Bonferroni significant group differences:

^a^ *versus* No ADHD group.

### Symptoms of ADHD

All participants were assessed with a self-report ADHD rating scale (ARS, see Materials). Based on their scores on the ARS, four groups were formed to use for group comparison analysis (see Data analyses). Group 1, named ‘*ADHD’* (n = 46), met the self-reported symptom criteria for both adulthood and childhood ADHD as defined in the DSM-5 (i.e., ≥ 5 symptoms of inattention and/or ≥ 5 symptoms of hyperactivity and impulsivity were reported to be present in the last six months and ≥ 6 symptoms of inattention and/or ≥ 6 symptoms of hyperactivity and impulsivity were reported to be present when 0–12 years old, respectively [1]). According to the presentation of current symptoms of ADHD, 43.5% (n = 20) of the ADHD group showed an inattentive presentation of ADHD, 21.7% (n = 10) a hyperactive/impulsive presentation of ADHD and 34.8% (n = 16) a mixed presentation of ADHD. Group 2, named ‘*Adult-only ADHD’* (n = 57), met the DSM-5 classification for adulthood ADHD, but not for childhood ADHD. Group 3, named ‘*Subthreshold ADHD’* (n = 162), did not meet the DSM-5 criteria for adulthood ADHD, but reported 3 or 4 current symptoms of inattention and/or 3 or 4 current symptoms of hyperactivity and impulsivity. Retrospective symptoms of ADHD (i.e., when 0–12 years old) were not part of the inclusion criteria for this group. However, 20.4% of participants in the Subthreshold ADHD group met the DSM-5 self-reported symptom criteria of childhood ADHD (S1 Table). Since the focus of the present study was on adults with current symptoms of ADHD, a non-persistent ADHD group (i.e., individuals who meet the self-reported DSM-5 symptom criteria for childhood ADHD but not for adulthood ADHD) was not taken into account. Finally, a matched control group was formed including participants classified as not having current ADHD symptoms (i.e., 0 or 1 current ADHD symptom(s) of inattention and 0 or 1 current ADHD symptom(s) of hyperactivity and impulsivity, irrespective of retrospective symptoms of ADHD). To control for age, sex and level of education, the 265 individuals of the ADHD, Adult-only ADHD and Subthreshold ADHD groups were matched with 265 participants without having current symptoms of ADHD, resulting in a matched ‘*No ADHD’* group (n = 265; Table 1). Participants who did not meet the matching criteria and/or inclusion criteria of one of the four groups (n = 762) were not taken into account when conducting group comparisons.

### Materials

#### ADHD rating scale

The ARS is a self-report questionnaire that was used to evaluate current and retrospective symptoms of ADHD [34,35]. The ARS was originally developed on the basis of the DSM-IV, however, can also be used with the current criteria as defined by the DSM-5 [1]. Both the versions for current and retrospective symptoms of the ARS have 23 items evaluating 18 symptoms of ADHD. On a four-point scale (0 = *‘rarely or never’*, 1 = *‘sometimes’*, 2 = *‘often’* and 3 = *‘very often’*), participants had to indicate for each item which answer alternative described their behavior best in the past six months (i.e., current symptoms) or during childhood when being 0–12 years old (i.e., retrospective symptoms). Using the scoring system of Kooij et al. [35], an ADHD symptom was classified as present when ‘often’ or ‘very often’ was answered on an item. Five criteria of ADHD as described in the DSM-5 (i.e., criteria 1a, 1d, 2a, 2d and 2e) were each represented by two items of the ARS and were classified as present when ‘often’ or ‘very often’ was answered on at least one of the two items. Total scores can be calculated based on all 23 items for both the current and retrospective version of the ARS. Furthermore, for each version, total scores can be calculated based on items representing symptoms of inattention (total of 11 items, *Cronbach’s alpha* = .86) and hyperactivity/impulsivity symptoms (total of 12 items, *Cronbach’s alpha* = .81 [34]). Higher scores are indicative of more (severe) symptoms of ADHD.

#### Impulsive buying questionnaire

The Impulsive Buying Questionnaire (IBQ) consists of 31 items focused on the tendency to buy on impulse [15,36]. Participants have to indicate on a four-point Likert scale (ranging from 1 = *‘strongly disagree’* to 4 = *‘strongly agree’*) whether they agree with the statements. Three components of impulsive buying can be evaluated based on the sum of specific items; a cognitive component (i.e., the thoughts and urge to buy on impulse; 15 items; e.g., *‘When I go shopping*, *I buy things that I did not intend to purchase’*; *Cronbach’s alpha* = .80), an affective component (i.e., the emotions and feelings that lead to impulsive behavior; 12 items; e.g., *‘I always buy it if I really like it’*; *Cronbach’s alpha* = .83) and a situational component (i.e., the available time and money that is needed to buy on impulse; 4 items; e.g., *‘I cannot afford unplanned purchases’*; *Cronbach’s alpha* = .09). A total score based on the sum of the cognitive and affective component can be calculated to evaluate the impulsive buying tendency of an individual (*Cronbach’s alpha* = .89). Higher scores represent a stronger tendency to buy on impulse.

#### Financial decision styles questionnaire

The Financial Decision Styles questionnaire (FDS) is used to evaluate five decision styles an individual can use when making financial decisions [15,36]. The FDS consists of 24 statements which differentiate a rational decision style (i.e., evaluating options before making a decision; 5 items; *Cronbach’s alpha* = .69), an intuitive decision style (i.e., relying on feelings and emotions when making a decision; 5 items; *Cronbach’s alpha* = .79), a dependent decision style (i.e., requiring the advice of others when making a decision; 5 items; *Cronbach’s alpha* = .82), an avoidant decision style (i.e., avoiding or postponing making a decision; 5 items; *Cronbach’s alpha* = .89), and a spontaneous decision style (i.e., being impulsive when making a decision; 4 items; *Cronbach’s alpha* = .74). For each statement, participants have to indicate to what extent a situation applies to them on a five-point scale (ranging from 1 = *‘strongly disagree’* to 5 = *‘strongly agree’*). Total scores are calculated as the sum of items related to a specific decision style. Higher scores indicate that a specific style is more frequently used.

#### Personal financial situation

Eight questions were asked to evaluate participants’ personal financial situation. Six questions required a yes-or-no response and focused on debts (i.e., *‘Do you have debts other than mortgage or study loans*?*’*), social security (i.e., *‘Do you receive social security*?*’*), saving money (i.e., *‘Do you have a savings account*?*’* and *‘Do you save actively*, *i*.*e*., *do you put money on your savings account on a regular basis*?*’*), saving for retirement (i.e., *‘Do/have you save(d) money for retirement*?*’*) and owning a house (i.e., *‘Do you own a house*?*’*). The annual gross income of participants was evaluated on a five-point scale (i.e., 1 = ‘*< €15*,*000’*, 2 = *‘€15*,*000–€25*,*000’*, 3 = *‘€25*,*000–€35*,*000’*, 4 = *‘€35*,*000–€45*,*000’*, and 5 = *‘> € 55*,*000’*). Finally, participants had to indicate the amount of money they retained each month after deduction of fixed expenses (in Euros). A second income (of, e.g., a partner) was not taken into account.

#### Personality

To evaluate personality, the NEO—Five Factor Inventory (NEO-FFI) was used [37]. With 60 items, the NEO-FFI measures the Big Five personality traits: neuroticism, extraversion, openness, agreeableness and conscientiousness. Participants have to indicate to what extent each item reflects their opinion on a five-point scale ranging from *‘strongly disagree’* to *‘strongly agree’*. For each scale, a maximum score of 60 can be obtained, with high scores indicating that a personality trait fits a person. The internal consistency of the Dutch version of the NEO-FFI is acceptable to good (*Cronbach’s alpha* = .57 to .88) and the inter-correlations of the scales are negligible to weak (*r* = .00 to .41 [38]).

#### Symptoms of depression

Symptoms of depression were evaluated using the Dutch version of the Beck Depression Inventory II (BDI-II-NL [39,40]). For each of the 21 items, participants have to indicate which out of four descriptions reflects their mood in the last two weeks (e.g., 0 = *‘I do not feel sad’*, 1 = *‘I feel sad’*, 2 = *‘I am sad all the time and I can’t snap out of it’* and 3 = *‘I am so sad or unhappy that I can’t stand it’*). Total scores range from 0 to 63 with higher scores indicating more symptoms and/or more severe symptoms of depression. The internal consistency of the BDI-II-NL is good (*Cronbach’s alpha* = .88 to .92) and the construct validity is strong (*r* = .79 to .85 [40]).

### Procedure and ethics

Data was collected between 2014 and 2019. Participants had to complete the questionnaires online which were distributed via Qualtrics [41]. Participants were recruited by using an online research panel and via the social networks of the researchers using social media, email or word of mouth. Participants recruited via the online research panel received a small financial compensation for participation, all other participants received no compensation. All participants had the Dutch nationality and were 18 years or older. Initially, 1475 participants were recruited for the study. Participation was voluntary and participants could withdraw from the study at any time. Consequently, 79 participants (5.4%) began but discontinued the questionnaire and were therefore not taken into account. Of the 79 participants who discontinued the study, 51.9% (n = 41) were from the online research panel and 48.1% (n = 38) of the participants were from the social network source. Since a financial compensation as received by the participants from the online research panel could serve as an external motivation, a validation check was performed in this group. Based on the performances on three validity scales of the Behavior Rating Inventory of Executive Function Adults (BRIEF-A [42,43]; i.e., negativity, infrequency and inconsistency), 5.0% of participants (n = 74) were excluded from the sample. Another 2.0% of participants (n = 30) were excluded from the sample because of other reasons (i.e., age below 18, age was unclear or no Dutch nationality). The remaining sample of 1292 participants were used in current study, of which 36.7% (n = 474) were recruited via the online research panel. By using unique ID codes, it was not possible for participants that were recruited via the research panel to repeat the study for extra financial compensation. Participants had to sign an informed consent prior to participation and were debriefed after completing the questionnaires. Questionnaires were presented in a fixed sequence to all participants (i.e., personal financial situation, IBQ, NEO-FFI, BDI-II-NL, FDS and ARS). For the participants in the research panel the BRIEF-A was added after the ARS. The study was approved by the Ethical Committee of Psychology of the University of Groningen, the Netherlands.

### Data analyses

#### Group comparisons

To examine group differences in impulsive buying and the use of financial decision styles, Analyses of Variance (ANOVA) have been conducted with the total score and the scores on the subscales of the IBQ and of the FDS as dependent variables, respectively. Post-hoc Bonferroni analyses were conducted when significant group differences were found. In addition, to evaluate group differences in participants’ personal financial situation, Pearson’s chi-square analyses were conducted for variables related to debts, receiving social security, saving money, saving for retirement and owning a house. Differences in income between groups were evaluated using a Kruskal-Wallis test and group differences on the variable ‘free money to spend’ were evaluated with an ANOVA. For all post-hoc analyses, effect sizes and 95% confidence intervals (95% CI) were calculated and interpreted as small (d = 0.20), medium (d = 0.50) and large (d = 0.80 [44]). Results were considered significant with an alpha of ≤ .05. Besides a Bonferroni correction in the post-hoc analyses, no further correction for alpha was applied, since current analyses partly replicate previous research in adults with ADHD [15] and smaller effects can be assumed because of the use of a community sample.

#### Predicting financial decision-making

To examine the relation between impulsive buying, the use of financial decision styles and symptoms of ADHD, and to determine the potential influence of personality and symptoms of depression on this relation, nine hierarchical linear regressions were performed in the whole sample (n = 1292) with the four IBQ and five FDS scores as dependent variables. First, to control for the potential effects of age (in years), sex (male/female), level of education [33] and annual income on impulsive buying and the use of financial decision styles, these independent variables were included in the first model (method: enter). To evaluate the potential effects of symptoms of depression and the Big Five personality traits, the total scores on the BDI and the five NEO-FFI personality traits, respectively, were included in the second model of the regression analyses (method: enter). Finally, the ARS total score reflecting current symptoms of ADHD was included in the third model. The ARS and BDI scores were LOG transformed to control for the strong positive skewness in these variables. After transformation, these variables showed a trend towards a normal distribution. *R*^*2*^/*sr*^*2*^ were used as measures of effect size and interpreted as small (*R*^*2*^/*sr*^*2*^ = below .08), medium (*R*^*2*^/*sr*^*2*^ = between .11 and .27) or large (*R*^*2*^/*sr*^*2*^ = above .30 [45]). When the ARS total score significantly contributed to the model, the hierarchical regression was repeated including the ARS total scores (i.e., current symptoms) of the inattentive items and the hyperactive/impulsive items separately. Because of the explorative approach of the regression analyses with the use of a relatively large number of independent variables, the inclusion of a relatively large sample and in order to control for multiple testing, a conservative alpha (p ≤ .001) was used for these analyses to reduce the change of overoptimism or type 1 errors.

## Results

### Group comparisons

#### Impulsive buying

Significant group differences were found on the IBQ total score and for both the cognitive and affective subscales of the IBQ (Table 2). The ADHD group obtained significantly higher scores on the IBQ cognitive subscale compared to both the No ADHD group (*Mean Difference (MD) = 2*.*7; Standard Error (SE)* = .88; *p* = .015; *d* = 0.54, 95% CI [0.22; 0.86]) and the Subthreshold ADHD group (*MD = 2*.*5; SE* = .92; *p* = .039; *d* = 0.40, 95% CI [0.07; 0.73]). Furthermore, the Adult-only ADHD group obtained significantly higher scores compared to the No ADHD group on the IBQ total score (*MD = 4*.*3; SE* = 1.5; *p* = .018; *d* = 0.47, 95% CI [0.18; 0.76]) and on both subscales (IBQ cognitive subscale: *MD = 2*.*2; SE* = .80; *p* = .033; *d* = 0.45, 95% CI [0.16; 0.73] and IBQ affective subscale: *MD = 2*.*1; SE* = .79; *p* = .046; *d* = 0.41, 95% CI [0.12; 0.70]). No significant differences were found regarding the other group comparisons and groups also did not differ in the situational subscale of the IBQ (Table 2).

**Table 2:** Impulsive buying and financial decision styles of total sample and ADHD groups.

	Total sample	ADHD	Adult-only ADHD	Subthreshold ADHD	No ADHD	*Group differences*		
						*Statistics*	*p*	*d*
**IBQ total score *M (SD)***	59.8 (10.0)	62.7 (10.5)	63.1 (11.3)^a^	59.6 (11.0)	58.8 (8.8)	F(3,529) = 4.4	.005*	0.316
**IBQ Cognitive component *M (SD)***	32.6 (5.5)	34.6 (6.3)^a^^,^^b^	34.2 (6.3)^a^	32.1 (6.1)	31.9 (4.7)	F(3,529) = 5.2	.001*	0.343
**IBQ Affective component *M (SD)***	27.4 (5.4)	28.1 (5.3)	28.9 (5.8)^a^	27.5 (5.9)	26.8 (5.0)	F(3,529) = 2.8	.039*	0.252
**IBQ Situational component *M (SD)***	9.7 (1.6)	9.9 (1.5)	9.7 (1.7)	9.6 (1.8)	9.7 (1.5)	F(3,529) = 0.6	.615	0.117
**FDS Rational *M (SD)***	18.8 (2.8)	18.4 (2.9)	18.5 (3.0)	18.9 (2.8)	18.8 (2.7)	F(3,529) = 0.7	.529	0.130
**FDS Intuitive *M (SD)***	16.4 (3.2)	16.9 (3.1)	17.0 (2.9)	16.6 (3.2)	16.1 (3.2)	F(3,529) = 1.9	.135	0.208
**FDS Dependent *M (SD)***	16.4 (3.6)	16.3 (3.6)	17.2 (3.4)	16.6 (3.8)	16.1 (3.6)	F(3,529) = 1.7	.161	0.196
**FDS Avoidant *M (SD)***	12.0 (4.0)	13.0 (4.5)^a^	14.5 (4.5)^a^^,^^b^	12.2 (4.1)	11.2 (3.5)	F(3,529) = 12.9	< .001*	0.541
**FDS Spontaneous *M (SD)***	8.7 (2.6)	9.9 (3.1)^a^^,^^b^	9.2 (2.9)	8.6 (2.5)	8.4 (2.3)	F(3,529) = 5.5	.001*	0.353

*Note*. ADHD = Attention Deficit Hyperactivity Disorder, IBQ = Impulsive Buying Questionnaire, FDS = Financial Decision Styles questionnaire.

* *p* ≤ .05. Post-hoc Bonferroni significant group differences:

^a^ *versus* No ADHD group and

^b^ *versus* Subthreshold ADHD group.

#### Financial decision styles

Significant group differences were found on the FDS avoidant subscale and the FDS spontaneous subscale (Table 2). For the FDS avoidant subscale, significantly higher scores were obtained by both the ADHD group and the Adult-only ADHD group compared to the No ADHD group (*MD = 1*.*8; SE* = .62; *p* = .030; *d* = 0.48, 95% CI [0.16; 0.79] and *MD = 3*.*3; SE* = .57; *p* < .001; *d* = 0.90, 95% CI [0.60; 1.19]; respectively). The scores on the FDS avoidant subscale of the Adult-only ADHD group were also significantly higher compared to the Subthreshold ADHD group (*MD = 2*.*3; SE* = .60; *p* = .001; *d* = 0.56, 95% CI [0.25; 0.86]). No significant differences were found on the FDS avoidant subscale between the other groups. With regard to the FDS spontaneous subscale, the ADHD group obtained significantly higher scores compared to the Subthreshold ADHD group (*MD = 1*.*3; SE* = .42; *p* = .009; *d* = 0.50, 95% CI [0.17; 0.83]) and the No ADHD group (*MD = 1*.*5; SE* = .40; *p* = .002; *d* = 0.60, 95% CI [0.28; 0.91]), while no significant differences were found between the other groups. Furthermore, groups did not differ with regard to the rational, intuitive and dependent subscales of the FDS.

#### Personal financial situation

Group differences were found with regard to actively saving money and saving for retirement (Table 3). In the ADHD group, only 19.6% saved for their retirement which were significantly less individuals than in the No ADHD group (41.5%; *X*^*2*^(1) = 8.0; *p* = .005; *d* = 0.32, 95% CI [0.13; 0.53]) and the Subthreshold ADHD group (37.0%; *X*^*2*^(1) = 4.9; *p* = .026; *d* = 0.31, 95% CI [0.06; 0.57]). Comparable results were found for the Adult-only ADHD group: 22.8% of the Adult-only ADHD group saved for their retirement, which were significantly less individuals compared to the No ADHD group (*X*^*2*^(1) = 7.0; *p* = .008; *d* = 0.30, 95% CI [0.06; 0.50]) and the Subthreshold ADHD group (*X*^*2*^(1) = 3.8; *p* = .050; *d* = 0.27, 95% CI [0.01; 0.52]). Furthermore, compared to the No ADHD group, the Adult-only ADHD group significantly less often saved money actively (38.8% of Adult-only ADHD group and 63.3% of No ADHD group saved actively, *X*^*2*^(1) = 10.3; *p* < .001; *d* = 0.38, 95% CI [0.14; 0.62]). Other group comparisons of these and other personal financial situation variables were not significant (e.g., annual year income, receiving social security or having debts; Table 3).

**Table 3:** Personal financial situation of the total sample and ADHD groups.

	Total sample	ADHD	Adult-only ADHD	Subthreshold ADHD	No ADHD	*Group differences*		
						*Statistics*	*p*	*d*
**‘What is approximately your annual gross income?’ *Mdn***	€25,000 to €35,000	< €15,000^1^	€15,000 to €25.000	€15,000 to €25.000	€15,000 to €25.000	*X*^*2*^(3) = 4.6	.206	0.110
**‘How much money can you approximately spend each month after deduction of fixed expenses?’**^1^ ***M (SD)***	€631.3 (€475.1)	€523.8 (€462.9)	€455.0 (€449.6)	€591.0 (€496.8)	€604.1 (441.0)	*F*(3,490) = 1.8	.145	0.211
**‘Do you receive social security?’*% yes***	33.0	30.4	31.6	30.9	26.0	*X*^*2*^(3) = 1.6	.663	0.110
**‘Do you have debts other than mortgage or study loans?’ *% yes***	7.7	8.7	3.5	9.9	5.3	*X*^*2*^(3) = 4.6	.202	0.187
**‘Do you have a savings account?’ *% yes***	91.1	87.0	86.0	92.0	94.7	*X*^*2*^(3) = 7.3	.065	0.236
**‘Do you save actively, i.e., do you put money on your savings account on a regular basis?’ *% yes***	58.8	55.0	38.8^a^	54.4	63.3	*X*^*2*^(3) = 11.2	.010*	0.308
**‘Do you save for your retirement?’ *% yes***	45.4	19.6^a^^,^^b^	22.8^a^^,^^b^	37.0	41.5	*X*^*2*^(3) = 13.2	.004*	0.320
**‘Do you own a house?’ *% yes***	61.6	37.0	35.1	41.4	50.2	*X*^*2*^(3) = 7.2	.066	0.236

*Note*. ADHD = Attention Deficit Hyperactivity Disorder.

^1^ Mode income is described since median income was between ‘< €15,000’ (n = 23) and ‘€15,000 to €25,000’ (n = 8).

^2^ Answering was not mandatory and outliers were excluded. Group size of the remaining samples: Total sample n = 1170, ADHD group n = 42, Adult-only ADHD group n = 55, Subthreshold ADHD group n = 144 and No ADHD group n = 253. Furthermore, income of (financial) partner was not taken into account.

* *p* ≤ .05. Post-hoc Bonferroni significant group differences:

^a^ *versus* No ADHD group and

^b^ *versus* Subthreshold ADHD group.

### Predicting financial decision-making

#### Impulsive buying behavior

In total, 26.6% of variance of the IBQ total score could be explained in the third regression model (*F*(11,1287) = 43.33; *p* < .001; Table 4). Symptoms of ADHD were, however, not a significant contributor to the model (Δ*F*(1,1276) = 4.0; *p* = .045; Δ*R*^*2*^ = .002). Instead, a significant positive relation was found with extraversion and neuroticism. Conscientiousness and agreeableness were significantly negatively related to the IBQ total score. Furthermore, being female and a lower age were also related to a stronger tendency to buy on impulse (Table 4).

**Table 4:** Hierarchical regressions of impulsive buying behavior in the total sample.

**IBQ total score**	**Model 1**				**Model 2**				**Model 3**			
	*B*	*ß*	*p*	*sr*^*2*^	*B*	*ß*	*p*	*sr*^*2*^	*B*	*ß*	*p*	*sr*^*2*^
**1**^**st**^ **block**												
Age	-0.17	-.34	< .001*	.094	-0.11	-.21	< .001*	.031	-0.10	-.20	< .001*	.027
Sex	3.16	.17	< .001*	.024	3.76	.20	< .001*	.029	3.79	.20	< .001*	.029
Level of education	-0.70	-.08	.005	.005	-0.35	-.04	.151	.001	-0.38	-.04	.123	.001
Income	-0.27	-.05	.112	.002	0.13	.02	.424	.000	0.12	.02	.479	.000
**2**^**nd**^ **block**												
Neuroticism					0.28	.23	< .001*	.025	0.26	.21	< .001*	.022
Extraversion					0.38	.25	< .001*	.043	0.37	.24	< .001*	.038
Openness					-0.06	-.04	.133	.001	-0.07	-.04	.113	.001
Agreeableness					-0.20	-.11	< .001*	.009	-0.18	-.10	< .001*	.007
Conscientiousness					-0.30	-.18	< .001*	.023	-0.28	-.16	< .001*	.020
Depression					1.21	.05	.089	.002	0.82	.03	.262	.001
**3**^**rd**^ **block**												
Symptoms of ADHD									1.80	.06	.045	.002
**Total *R***^***2***^ **adjusted**	15.6%, *p* < .001*				26.4%, *p* < .001*				26.6%, *p* < .001*			
**Δ*R***^***2***^					11.4%, *p* < .001*				0.2%, *p* = .045			
**IBQ cognitive**	**Model 1**				**Model 2**				**Model 3**			
	*B*	*ß*	*p*	*sr*^*2*^	*B*	*ß*	*p*	*sr*^*2*^	*B*	*ß*	*p*	*sr*^*2*^
**1**^**st**^ **block**												
Age	-0.08	-.30	< .001*	.075	-0.05	-.17	< .001*	.021	-0.05	-.16	< .001*	.018
Sex	1.14	.11	< .001*	.011	1.87	.18	< .001*	.024	1.89	.18	< .001*	.024
Level of education	-0.30	-.06	.033	.003	-0.09	-.02	.492	.000	-0.10	-.02	.446	.000
Income	-0.07	-.02	.457	.000	0.18	.06	.045	.002	0.18	.06	.052	.002
**2**^**nd**^ **block**												
Neuroticism					0.11	.16	< .001*	.012	0.10	.15	< .001*	.010
Extraversion					0.19	.23	< .001*	.036	0.18	.22	< .001*	.032
Openness					-0.04	-.05	.057	.002	-0.05	-.05	.049	.002
Agreeableness					-0.13	-.14	< .001*	.013	-0.12	-.13	< .001*	.011
Conscientiousness					-0.25	-.27	< .001*	.058	-0.25	-.27	< .001*	.052
Depression					0.20	.02	.611	.000	0.04	.00	.915	.000
**3**^**rd**^ **block**												
Symptoms of ADHD									0.74	.05	.137	.002
**Total *R***^***2***^ **adjusted**	10.3%, *p* < .001*				23.3%, *p* < .001*				23.3%, *p* < .001*			
**Δ*R***^***2***^					13.3%, *p* < .001*				0.1%, *p* = .137			
**IBQ affective**	**Model 1**				**Model 2**				**Model 3**			
	*B*	*ß*	*p*	*sr*^*2*^	*B*	*ß*	*p*	*sr*^*2*^	*B*	*ß*	*p*	*sr*^*2*^
**1**^**st**^ **block**												
Age	-0.09	-.32	< .001*	.084	-0.06	-.21	< .001*	.032	-0.06	-.20	< .001*	.027
Sex	2.00	.19	< .001*	.033	1.87	.18	< .001*	.023	1.89	.18	< .001*	.024
Level of education	-0.40	-.08	.003	.006	-0.25	-.05	.062	.002	-0.27	-.06	.048	.002
Income	-0.20	-.06	.032	.003	-0.05	-.02	.574	.000	-0.06	.02	.509	.000
**2**^**nd**^ **block**												
Neuroticism					0.17	.26	< .001*	.033	0.17	.24	< .001*	.029
Extraversion					0.20	.23	< .001*	.037	0.18	.22	< .001*	.032
Openness					-0.02	-.02	.430	.000	-0.02	-.02	.379	.000
Agreeableness					-0.07	-.07	.009	.004	-0.06	-.06	.033	.003
Conscientiousness					-0.04	-.05	.101	.002	-0.03	-.03	.229	.001
Depression					1.03	.08	.009	.004	0.80	.06	.051	.000
**3**^**rd**^ **block**												
Symptoms of ADHD									1.07	.07	.032	.008
**Total *R***^***2***^ **adjusted**	15.8%, *p* < .001*				24.0%, *p* < .001*				24.2%, *p* < .001*			
**Δ*R***^***2***^					8.5%, *p* < .001*				0.3%, *p* = .032			
**IBQ situational**	**Model 1**				**Model 2**				**Model 3**			
	*B*	*ß*	*p*	*sr*^*2*^	*B*	*ß*	*p*	*sr*^*2*^	*B*	*ß*	*p*	*sr*^*2*^
**1**^**st**^ **block**												
Age	0.01	.06	.065	.003	0.01	.07	.041	.003	0.01	.08	.023	.004
Sex	-0.24	-.08	.009	.005	-0.08	-.03	.409	.001	-0.08	-.03	.435	.000
Level of education	0.07	.05	.112	.002	0.10	.07	.033	.003	0.09	.07	.039	.003
Income	0.05	.05	.101	.002	0.06	.07	.050	.003	0.06	.06	.057	.003
**2**^**nd**^ **block**												
Neuroticism					-0.02	-.10	.014	.005	-0.02	-.11	.008	.005
Extraversion					0.01	.03	.354	.001	0.01	.02	.521	.000
Openness					-0.02	-.06	.036	.003	-0.02	-.06	.031	.003
Agreeableness					-0.01	-.02	.567	.000	-0.00	-.01	.762	.000
Conscientiousness					-0.04	-.14	< .001*	.016	-0.04	-.14	< .001*	.014
Depression					-0.09	-.02	.501	.000	-0.13	-.04	.322	.001
**3**^**rd**^ **block**												
Symptoms of ADHD									0.21	.05	.192	.001
**Total *R***^***2***^ **adjusted**	1.6%, *p* < .001*				3.5%, *p* < .001*				3.6%, *p* < .001*			
**Δ*R***^***2***^					2.4%, *p* < .001*				0.1%, *p* = .192			

*Note*. ADHD = Attention Deficit Hyperactivity Disorder, IBQ = Impulsive Buying Questionnaire. Neuroticism, extraversion, openness, agreeableness and conscientiousness are measured with the Neuroticism-Extraversion-Openness Five Factor Inventory (NEO-FFI). Symptoms of depression are measured with the Beck Depression Inventory II (BDI-II-NL). Symptoms of ADHD are measured with the Attention Deficit Hyperactivity Disorder rating scale (ARS) current version.

* p ≤ .001 is considered significant.

With regard to the cognitive, affective and situational components of the IBQ, 23.3% (*F*(11,1288) = 36.7; *p* < .001); 24.2% (*F*(11,1288) = 38.4; *p* < .001) and 3.6% (*F*(11,1289) = 5.4; *p* < .001) of variance could be explained in the third regression model, respectively (Table 4). Symptoms of ADHD, again, did not significantly contribute to these models (cognitive: Δ*R*^*2*^ = .001; Δ*F*(1,1277) = 2.2; *p* = .137; affective: Δ*R*^*2*^ = .003; *F*(Δ*F*(1,1277) = 4.6; *p* = .032; and situational: Δ*R*^*2*^ = .001; *F*(Δ*F*(1,1278) = 1.7; *p* = .192). For the IBQ cognitive component, the significant predictors were similar to the contributors of the IBQ total score: while extraversion and neuroticism were significantly positively related, conscientiousness and agreeableness were significantly negatively related to the cognitive component. Also, being female and a lower age were significantly related to higher scores on the cognitive component of impulsive buying. For the IBQ affective component, extraversion and neuroticism were found to be significantly positively related, while other Big Five personality traits and symptoms of depression did not significantly contribute to this model. However, being female and a lower age were significantly related to the affective component of impulsive buying as well. Regarding the situational component of the IBQ, only conscientiousness was found to be a significant negative predictor (Table 4).

#### Financial decision styles

Symptoms of ADHD did not significantly contribute to the third model of any of the five FDS decision styles (rational: Δ*R*^*2*^ = .000; Δ*F*(1,1278) = 0.3; *p* = .580; intuitive: Δ*R*^*2*^ = .000; Δ*F*(1,1278) = 0.1; *p* = .827; dependent: Δ*R*^*2*^ = .006; Δ*F*(1,1278) = 8.7; *p* = .003; avoidant: Δ*R*^*2*^ = .005; Δ*F*(1,1278) = 7.6; *p* = .006 and spontaneous: Δ*R*^*2*^ = .001; Δ*F*(1,1278) = 1.2; *p* = .278). Decision styles could, however, be explained by other variables included in the regression model (Table 5). In total, 21.0% of variance of the FDS rational subscale could be explained in the third regression model (*F*(11,1289) = 32.2; *p* < .001). The use of a rational decision style was significantly positively related to conscientiousness and openness and significantly negatively related to extraversion. In addition, level of education was also a significant positive predictor of the rational decision style (Table 5). With regard to the FDS intuitive subscale, 9.8% of variance could be explained in the third regression model (*F*(11,1289) = 13.7; *p* < .001). The use of an intuitive decision style was significantly negatively related to level of education and significantly positively related to extraversion and symptoms of depression (Table 5). The third regression model of the FDS dependent subscale was also significant (*R*^*2*^ = .115; *F*(11,1289) = 16.3; *p* < .001) and the use of a dependent decision style was significantly positively related to neuroticism, extraversion and being female (Table 5). With regard to the FDS avoidant subscale, 23.6% of variance could be explained in the third regression model (*F*(11,1289) = 37.2; *p* < .001). The use of an avoidant decision style was significantly positively related to age, neuroticism and extraversion and significantly negatively related to conscientiousness and agreeableness (Table 5). Finally, 24.0% of variance could be explained in the third regression model of the FDS spontaneous subscale (*F*(11,1289) = 37.9; *p* < .001). The use of a spontaneous decision style was significantly positively related to extraversion and neuroticism and significantly negatively related to conscientiousness and agreeableness (Table 5).

**Table 5:** Hierarchical regressions of financial decision-making styles in the total sample.

**FDS rational**	**Model 1**				**Model 2**				**Model 3**			
	*B*	*ß*	*p*	*sr*^*2*^	*B*	*ß*	*p*	*sr*^*2*^	*B*	*ß*	*p*	*sr*^*2*^
**1**^**st**^ **block**												
Age	0.02	.11	< .001*	.010	0.00	.01	.659	.000	0.00	.02	.583	.000
Sex	0.15	.03	.325	.001	-0.23	-.05	.123	.001	-0.23	-.05	.128	.001
Level of education	0.51	.21	< .001*	.038	0.34	.14	< .001*	.015	0.34	.14	< .001*	.015
Income	0.17	.11	.001*	.008	0.02	.02	.615	.000	0.02	.01	.633	.000
**2**^**nd**^ **block**												
Neuroticism					-0.03	-.08	.020	.003	-0.03	-.09	.017	.003
Extraversion					-0.06	-.15	< .001*	.016	-0.07	-.16	< .001*	.016
Openness					0.07	.17	< .001*	.024	0.07	.17	< .001*	.024
Agreeableness					-0.00	-.00	.939	.000	0.00	.00	.973	.000
Conscientiousness					0.17	.38	< .001*	.108	0.17	.38	< .001*	.105
Depression					0.45	.07	.025	.003	0.42	.06	.043	.003
**3**^**rd**^ **block**												
Symptoms of ADHD									0.14	.02	.580	.000
**Total *R***^***2***^ **adjusted**	0.7%, *p* < .001*				21.1%, *p* < .001*				21.0%, *p* < .001*			
**Δ*R***^***2***^					14.7%, *p* < .001*				0.0%, *p* = .580			
**FDS intuitive**	**Model 1**				**Model 2**				**Model 3**			
	*B*	*ß*	*p*	*sr*^*2*^	*B*	*ß*	*p*	*sr*^*2*^	*B*	*ß*	*p*	*sr*^*2*^
**1**^**st**^ **block**												
Age	0.01	.04	.239	.001	0.01	.07	.028	.003	0.01	.07	.036	.003
Sex	0.37	.06	.050	.003	0.29	.05	.150	.001	0.29	.05	.152	.001
Level of education	-0.65	-.21	< .001*	.038	-0.59	-.19	< .001*	.028	-0.58	-.19	< .001*	.027
Income	-0.18	-.09	.004	.005	-0.20	-.10	.002	.007	-0.20	-.10	.002	.007
**2**^**nd**^ **block**												
Neuroticism					0.03	.06	.104	.002	0.03	.06	.101	.002
Extraversion					0.09	.17	< .001*	.020	0.09	.17	< .001*	.020
Openness					-0.02	-.03	.320	.001	-0.02	-.03	.325	.001
Agreeableness					-0.04	-.07	.029	.003	-0.04	-.07	.029	.003
Conscientiousness					0.04	.06	.037	.003	0.04	.06	.045	.003
Depression					1.02	.12	< .001*	.010	1.04	.12	< .001*	.009
**3**^**rd**^ **block**												
Symptoms of ADHD									-0.08	-.01	.827	.000
**Total *R***^***2***^ **adjusted**	6.9%, *p* < .001*				9.9%, *p* < .001*				9.8%, *p* < .001*			
**Δ*R***^***2***^					3.4%, *p* < .001*				0.0%, *p* = .827			
**FDS dependent**	**Model 1**				**Model 2**				**Model 3**			
	*B*	*ß*	*p*	*sr*^*2*^	*B*	*ß*	*p*	*sr*^*2*^	*B*	*ß*	*p*	*sr*^*2*^
**1**^**st**^ **block**												
Age	-0.03	-.17	< .001*	.024	-0.02	-.08	.008	.005	-0.01	-.06	.049	.003
Sex	1.12	.16	< .001*	.021	0.78	.11	.001*	.008	0.80	.11	< .001*	.009
Level of education	-0.11	-.03	.254	.001	-0.10	-.03	.350	.001	-0.11	-.03	.278	.001
Income	-0.06	-.03	.412	.000	0.02	.01	.786	.000	0.01	.00	.889	.000
**2**^**nd**^ **block**												
Neuroticism					0.13	.27	< .001*	.035	0.12	.25	< .001*	.029
Extraversion					0.12	.20	< .001*	.029	0.11	.18	< .001*	.023
Openness					0.01	.01	.650	.000	0.01	.01	.737	.000
Agreeableness					0.03	.05	.101	.002	0.05	.07	.028	.003
Conscientiousness					-0.03	-.04	.146	.001	-0.02	-.03	.387	.001
Depression					-0.33	-.04	.271	.001	-0.56	-.06	.067	.002
**3**^**rd**^ **block**												
Symptoms of ADHD									1.10	.10	.003	.006
**Total *R***^***2***^ **adjusted**	5.7%, *p* < .001*				11.0%, *p* < .001*				11.5%, *p* < .001*			
**Δ*R***^***2***^					5.7%, *p* < .001*				0.6%, *p* = .003			
**FDS avoidant**	**Model 1**				**Model 2**				**Model 3**			
	*B*	*ß*	*p*	*sr*^*2*^	*B*	*ß*	*p*	*sr*^*2*^	*B*	*ß*	*p*	*sr*^*2*^
**1**^**st**^ **block**												
Age	-0.01	-.05	.099	.002	0.02	.09	.002	.006	0.02	.11	< .001*	.008
Sex	-0.22	-.03	.328	.001	0.22	.03	.303	.001	0.24	.03	.261	.001
Level of education	-0.26	-.07	.015	.004	-0.09	-.03	.361	.001	-0.10	-.03	.292	.001
Income	-0.33	-.14	< .001*	.015	-0.04	-.02	.597	.000	-0.04	-.02	.513	.000
**2**^**nd**^ **block**												
Neuroticism					0.15	.29	< .001*	.043	0.14	.28	< .001*	.036
Extraversion					0.09	.14	< .001*	.014	0.08	.12	< .001*	.010
Openness					-0.03	-.04	.106	.002	-0.03	-.05	.083	.002
Agreeableness					-0.09	-.13	< .001*	.012	-0.08	-.11	< .001*	.008
Conscientiousness					-0.20	-.29	< .001*	.067	-0.19	-.28	< .001*	.058
Depression					-0.01	-.00	.966	.000	-0.23	-.02	.449	.000
**3**^**rd**^ **block**												
Symptoms of ADHD									1.00	.08	.006	.005
**Total *R***^***2***^ **adjusted**	2.9%, *p* < .001*				23.2%, *p* < .001*				23.6%, *p* < .001*			
**Δ*R***^***2***^					20.6%, *p* < .001*				0.5%, *p* = .006			
**FDS spontaneous**	**Model 1**				**Model 2**				**Model 3**			
	*B*	*ß*	*p*	*sr*^*2*^	*B*	*ß*	*p*	*sr*^*2*^	*B*	*ß*	*p*	*sr*^*2*^
**1**^**st**^ **block**												
Age	-0.03	-.24	< .001*	.049	-0.01	-.10	.001*	.006	-0.01	-.09	.003	.005
Sex	-0.27	-.06	.048	.003	0.32	.07	.019	.003	-0.33	-.07	.017	.003
Level of education	-0.11	-.05	.098	.002	0.01	.01	.819	.000	-0.01	-.01	.862	.000
Income	-0.05	-.04	.232	.001	0.07	.05	.123	.001	-0.06	-.04	.136	.001
**2**^**nd**^ **block**												
Neuroticism					0.04	.13	< .001*	.009	0.04	.13	< .001*	.008
Extraversion					0.09	.23	< .001*	.036	0.09	.22	< .001*	.032
Openness					-0.03	-.07	.009	.004	-0.03	-.07	.008	.004
Agreeableness					-0.12	-.26	< .001*	.048	-0.11	-.25	< .001*	.044
Conscientiousness					-0.13	-.30	< .001*	.068	-0.12	-.29	< .001*	.063
Depression					-0.12	-.02	.512	.000	-0.17	-.03	.361	.001
**3**^**rd**^ **block**												
Symptoms of ADHD									0.25	.03	.278	.001
**Total *R***^***2***^ **adjusted**	6.0%, *p* < .001*				24.0%, *p* < .001*				24.0%, *p* < .001*			
**Δ*R***^***2***^					18.3%, *p* < .001*				0.0%, *p* = .278			

*Note*. ADHD = Attention Deficit Hyperactivity Disorder, FDS = financial decision-making styles questionnaire. Neuroticism, extraversion, openness, agreeableness and conscientiousness are measured with the Neuroticism-Extraversion-Openness Five Factor Inventory (NEO-FFI). Symptoms of depression are measured with the Beck Depression Inventory II (BDI-II-NL). Symptoms of ADHD are measured with the Attention Deficit Hyperactivity Disorder rating scale (ARS) current version.

* p ≤ .001 is considered significant.

## Discussion

The primary goal of the present study was to further investigate the influence of current symptoms of ADHD on two types of financial performance, i.e., impulsive buying and financial decision styles which are of influence on the capability to make financial decisions [11]. Furthermore, it was investigated to what extent personality and symptoms of depression are of influence on the association between impulsive buying, financial decision styles and symptoms of ADHD. Based on a recent study [15], symptoms of ADHD were expected to be related to impulsive buying, in particular to the cognitive component of impulsive buying. Furthermore, the use of an avoidant or a spontaneous decision style in situations requiring financial decisions, which are both negatively related to decision-making [32], were expected to be related to symptoms of ADHD.

In the present study, the tendency to buy on impulse and/or components of impulsive buying were indeed stronger in individuals experiencing symptoms of ADHD in adulthood (i.e., the ADHD group and Adult-only ADHD group) compared to individuals without symptoms of ADHD. Furthermore, individuals who met the self-reported symptom criteria for ADHD (i.e., the ADHD group) showed higher scores on the cognitive component of impulsive buying compared to individuals without symptoms of ADHD and individuals with current subthreshold symptoms of ADHD. A similar result was found for individuals in the Adult-only ADHD group compared to individuals without symptoms of ADHD (i.e., the No ADHD group). Previous research reported a significant relation between current symptoms of ADHD and compulsive buying, a pathological form of impulsive buying [46,47], particularly with ADHD symptoms of hyperactivity and impulsivity [46]. In the regression analyses, however, no association between symptoms of ADHD and impulsive buying was found. In contrast, the results of the present study suggest that personality traits seem to play a role when buying on impulse. The cognitive, affective and combined component (i.e., total score) of impulsive buying were found to be positively related to traits of neuroticism and extraversion and negatively related to traits of conscientiousness and agreeableness, with the exception of the affective component which was not related to conscientiousness and agreeableness. Comparable findings were found in previous research on impulsive buying and personality within a non-clinical adult sample [30,31]. Although the strength of relations found was weak, in previous and present research the strongest associations with impulse buying were most consistently found for extraversion and neuroticism: Individuals who are strongly focused on and engaged in the external environment (i.e., high on extraversion) and/or individuals who experience a strong tendency of emotional instability or negative emotions (i.e., high on neuroticism) tend to have a higher tendency to buy on impulse compared to individuals with low levels of these personality traits. In accordance with previous literature [15,31], being female and a younger age were also associated with a stronger tendency to buy on impulse. Although the ADHD groups did not statistically differ with regard to age and sex, individuals in the ADHD group seem to be slightly younger compared to the other groups when focusing on the age range of these groups. This observation needs to be taken into account when interpreting these results. The situational component of impulsive buying, i.e., having enough time and money to make an impulsive purchase, was not associated with symptoms of ADHD and could also not be explained by demographic variables, symptoms of depression and the majority of personality traits. Only conscientiousness was significantly negatively related with the situational component. It is, however, important to keep in mind that the internal consistency of this component of the IBQ was very weak.

The results with regard to the use of specific financial decision styles showed a similar pattern as found for impulse buying. In accordance with previous research [15], significant group differences were found between the ADHD group and No ADHD group on the avoidant and spontaneous decision styles. The ADHD group also more often used a spontaneous decision style compared to the Subthreshold ADHD group. Furthermore, compared to the No ADHD group and Subthreshold ADHD group, an avoidant decision style was more often used by the Adult-only group. Symptoms of ADHD, however, could not predict the scores on the avoidant and spontaneous decision styles in the regression analyses. Instead, personality traits seem to explain a significant amount of variance of financial decision styles. The avoidant and spontaneous decision styles were found to be significantly positively related to traits of neuroticism and extraversion and significantly negatively related to traits of conscientious and agreeableness. The use of other financial decision styles was also not associated with symptoms of ADHD but was, besides personality traits, related to symptoms of depression (i.e., intuitive style), level of education (i.e., rational and intuitive style) and sex (i.e., dependent style).

When interpreting the results regarding impulsive buying and the use of financial decision styles, it is important to keep in mind that associations were found between symptoms of ADHD and personality traits (S1 and S2 Tables). ADHD symptoms seem to be specifically associated with high neuroticism and low conscientiousness and agreeableness. Previous research found that conscientiousness appears more strongly related to inattentive symptoms than to hyperactive/impulsive symptoms (see Gomez and Corr [26] for a review and meta-analyses). The presence of significant group differences between the ADHD groups on financial decision-making leads to the expectation of an association between symptoms of ADHD and financial decision-making. However, the associations that were found between ADHD symptoms and personality traits (S1 and S2 Tables) might, at least partly, explain the absence of an association between symptoms of ADHD and financial decision-making in the regression analyses when controlling for demographic characteristics, symptoms of depression and personality traits. Interestingly, in the present study, an association between extraversion and both impulsive buying and the use of specific financial decision styles is most consistently found, however, no association has been found between extraversion and symptoms of ADHD (S1 and S2 Tables).

With regard to the personal financial situation, the ADHD group less often saved for their retirement compared to the No ADHD group. On all other aspects (e.g., income, debts, having a savings account) the financial situation of the ADHD group was comparable to individuals without symptoms of ADHD. Compared to the No ADHD group, the Adult-only ADHD group also made less often financial decisions for the future (i.e., they less often saved money for their retirement and less often used their savings account). Previous research comparing a clinical ADHD group with healthy controls, however, revealed that adults with ADHD have various problems related to their personal financial situation (e.g., more often debts, exceeding credit card loans or less often saving money [4,15]). The discrepancy between the current and previous studies could be explained by the fact that previous studies included clinical samples which most likely consisted of adults with ADHD with more (severe) ADHD symptoms and problems in daily life than participants with symptoms of ADHD in a community sample.

Besides a group meeting the self-reported DSM-5 symptom criteria of adult ADHD (i.e., ADHD group), two other ADHD groups were created based on the number of reported symptoms of ADHD: an Adult-only ADHD group meeting the self-reported symptom criteria of adult ADHD but not for childhood ADHD and a Subthreshold ADHD group reporting a subthreshold number of current symptoms of ADHD (i.e., 3 or 4 symptoms of either inattention or hyperactivity/impulsivity). Based on the results, no evidence has been found that individuals with a subthreshold number of ADHD symptoms have difficulties with financial decision-making, since no differences were found between the Subthreshold ADHD group and the No ADHD group with regard to impulsive buying and the use of financial decision styles. However, the Adult-only ADHD group showed inadequate financial performances on both the cognitive and affective domain of impulsive buying. Furthermore, the avoidant style was more often used by this ADHD group compared to individuals without symptoms of ADHD. This indicates that individuals with current symptoms of ADHD without the presence of retrospective symptoms of ADHD might have difficulties with financial decision-making, similar to individuals meeting the self-reported symptom criteria of adult ADHD. Previous research already found similar neuropsychological performances and personality profiles in individuals who met all DSM-IV criteria for childhood-onset ADHD and individuals having so-called late-onset ADHD [48,49]. These findings, and the findings in the present study, support the current discussion about the age of onset of ADHD and the possibility of a late-onset form of adult ADHD [50].

### Limitations

An important limitation that needs to be taken into account when interpreting the results of the present study is the lack of clinical information about the participants (e.g., about current or previous diagnosis of ADHD). Consequently, results may not be generalized to a clinical ADHD population. Current findings with regard to impulsive buying and the use of financial decision styles are, however, comparable with and seem to confirm previous research using a clinical group of adults with ADHD [15]. Furthermore, comorbidities, such as other (psychiatric) disorders (with the exception of symptoms of depression), and the use of medication were not taken into account in the present study. In a previous study, however, no evidence has been found that comorbidities are of influence on the financial decision-making capabilities of individuals with ADHD [15]. The influence of current and previous use of medication on the capability to make financial decisions is unknown. In present study, only a small number of participants (i.e., 4.3%) used either antidepressants, antipsychotics, benzodiazepines or stimulants. Therefore, the influence of these medication in current results is probably negligible. The use of other types of medications was not sufficiently studied in current study.

Another limitation is that the total administration time of the questionnaires was longer than initially expected. On average, participants needed 56 minutes to complete all questionnaires. The sequence of questionnaires was, however, equal for all participants (see Materials and Methods). Nevertheless, especially for individuals with attentional problems, this duration may have been of influence on the pattern of answers on the questionnaires. Participants were able to pause the program and complete the questionnaires at a later point in time, which on the one hand might have reduced the effects of inattention and fatigue, but, on the other hand, also contributed to the relatively long administration time of the questionnaires. In this respect, it is interesting that the ADHD groups did not differ in the average administration time (*F*(3,506) = .33; *p* = .808). A third limitation is that the use of different recruitment and compensation modalities might have resulted in differences in clinical or demographical characteristics and motivation between participants recruited via an online research panel (n = 474) and participants recruited via other modalities (n = 818). Additional analyses comparing participants recruited via these two modalities, however, indicated no significant differences between groups with regard to retrospective and current symptoms of ADHD. Furthermore, no marked differences in the main findings were found between these modalities (data not published).

Another limitation is that no direct causal conclusions can be drawn about the observed associations between the dependent and independent variables in current study. This is a common problem in cross-sectional studies and especially when studying personality and psychopathology [51,52]. In the current study, it is conceivable that there is an overlap in the studied constructs of personality, impulsive buying and decision styles, that these constructs simultaneously influence each other or that these constructs share an underlying etiology [51]. Prospective longitudinal research is, therefore, recommended to further investigate the causality between personality and financial decision styles and impulsive buying. A final limitation is that the use of a conservative alpha level for the regression analyses reduced the risk of type 1 errors but increased the possibility of type 2 errors. Furthermore, the use of a large sample with a relatively small percentage of individuals with symptoms of ADHD or individuals meeting the self-reported symptom criteria of adult ADHD in the regression analyses, may have contributed to potential type 2 errors. All relations found in the present study were, however, mostly weak which indicates a low likelihood of type 2 errors. Furthermore, additional regression analyses using only participants from the four ADHD groups (n = 529; data not published) showed again no significant association between financial decision-making and symptoms of ADHD, while results with regard to the relation with demographic variables, personality or symptoms of depression were comparable to the original analyses.

## Conclusion

Despite the described limitations, the results of the present study appear to confirm previous research in adults with ADHD and indicate more impulsive buying and more frequent use of disadvantageous financial decisions styles (i.e., avoidant and spontaneous styles) in individuals fulfilling self-reported current ADHD criteria (i.e., individuals with ADHD and adult-only or late-onset ADHD) compared to individuals without symptoms of ADHD. Contextual factors, i.e., personality and demographic variables, were also found to be related to both impulsive buying and the use of specific financial decision styles. Personality and demographic variables might (at least partly) be of influence on the association between impulsive buying, the use of financial decision styles and ADHD since no significant association between symptoms of ADHD and these types of financial performance could be found when controlling for personality characteristics, symptoms of depression and demographics. However, no direct causal conclusions can be drawn and prospective longitudinal research is suggested to further investigate causality between these variables. The evaluation of financial decision-making in adults with ADHD remains nevertheless a topic of major interest in both research and clinical practice, since problems with managing financial matters may have far-reaching consequences for both patients and their social environment, and more attention for this topic is therefore recommended.

## Supporting information

S1 Table Symptoms of ADHD, personality and symptoms of depression of the total sample and ADHD groups. (DOCX)

S2 Table Pearson correlations in the total sample. (DOCX)
