# Supplementary material for: Financial decision-making in a community sample of adults with and without current symptoms of ADHD
Source: PLoS One. 2020 Oct 12;15(10):e0239343. doi: 10.1371/journal.pone.0239343 (PMC7549773; doi:10.1371/journal.pone.0239343)
Supplement: S1 Table — (DOCX) [file pone.0239343.s001.docx]

**S1 Table. Symptoms of ADHD, personality and symptoms of depression of the total sample and ADHD groups.**

|  | **Total sample** | **ADHD** | **Adult-only ADHD** | **Subthreshold ADHD** | **No ADHD** | *Group differences* | | |
| --- | --- | --- | --- | --- | --- | --- | --- | --- |
|  |  |  |  |  |  | *Statistics* | *p* | *d* |
| **Current symptoms of ADHD *M (SD)*** | 13.1 (8.6) | 34.5 (8.1)^a,b,c^ | 29.8 (7.3)^a,b^ | 20.7 (4.9)^a^ | 10.3 (5.4) | F(3,529) = 376.2 | < .001* | 2.922 |
| DSM-5 ADHD classification^1^ *%* | 7.9 | 100 | 100 | 0.0 | 0.0 |  |  |  |
| **Retrospective symptoms of ADHD *M (SD)*** | 13.8 (11.7) | 40.4 (8.1)^a,b,c^ | 19.5 (9.3)^a^ | 20.1 (11.1)^a^ | 12.1 (10.0) | F(3,529) = 108.1 | < .001* | 1.566 |
| DSM-5 ADHD classification^2^ *%* | 10.1 | 100 | 0.0 | 20.4 | 6.7 |  |  |  |
| **Neuroticism *M (SD)*** | 29.9 (7.6) | 36.1 (7.2)^a^ | 38.3 (7.6)^a,b^ | 33.1 (7.9)^a^ | 29.6 (6.4) | F(3,529) = 31.7 | < .001* | 0.848 |
| Very low (*%* stanine ≤ 1) | 5.1 | 2.2 | 0.0 | 3.1 | 3.0 |  |  |  |
| Very high (*%* stanine ≥ 9) | 2.1 | 4.3 | 12.3 | 5.0 | 0.8 |  |  |  |
| **Extraversion *M (SD)*** | 40.1 (6.1) | 40.3 (7.2) | 38.5 (7.3) | 40.4 (7.4) | 41.0 (5.6) | F(3,529) = 2.3 | .075 | 0.229 |
| Very low (*%* stanine ≤ 1) | 3.5 | 6.5 | 3.6 | 6.2 | 1.2 |  |  |  |
| Very high (*%* stanine ≥ 9) | 2.8 | 2.2 | 8.8 | 5.0 | 3.0 |  |  |  |
| **Openness *M (SD)*** | 36.9 (5.8) | 37.5 (6.9) | 37.3 (5.0) | 37.0 (6.2) | 36.8 (5.8) | F(3,529) = 0.3 | .827 | 0.083 |
| Very low (*%* stanine ≤ 1) | 1.6 | 2.2 | 0.0 | 2.4 | 1.0 |  |  |  |
| Very high (*%* stanine ≥ 9) | 2.9 | 8.6 | 1.8 | 3.7 | 2.7 |  |  |  |
| **Agreeableness *M (SD)*** | 44.0 (5.2) | 38.9 (6.3)^a,b^ | 41.1 (6.2)^a^ | 42.7 (5.5)^a^ | 44.3 (5.1) | F(3,529) = 16.4 | < .001* | 0.610 |
| Very low (*%* stanine ≤ 1) | 4.0 | 30.4 | 14.1 | 6.2 | 2.3 |  |  |  |
| Very high (*%* stanine ≥ 9) | 3.1 | 2.2 | 0.0 | 1.8 | 4.9 |  |  |  |
| **Conscientiousness *M (SD)*** | 44.6 (5.5) | 39.7 (5.7)^a,b^ | 39.5 (6.2)^a,b^ | 44.0 (6.0) | 44.7 (5.2) | F(3,529) = 21.5 | < .001* | 0.698 |
| Very low (*%* stanine ≤ 1) | 5.1 | 23.9 | 22.8 | 6.8 | 3.7 |  |  |  |
| Very high (*%* stanine ≥ 9) | 4.2 | 0.0 | 0.0 | 4.3 | 4.2 |  |  |  |
| **Symptoms of depression *M (SD)*** | 5.9 (6.4) | 12.1 (9.8)^a,b^ | 13.5 (10.1)^a,b^ | 8.2 (8.1)^a^ | 3.8 (4.2) | F(3,529) = 45.7 | < .001* | 1.018 |
| No to minimal (*%* score 0 – 13) | 90.6 | 71.7 | 57.9 | 84.0 | 96.6 |  |  |  |
| Mild (*%* score 14 – 19) | 5.3 | 4.3 | 22.8 | 8.0 | 2.6 |  |  |  |
| Moderate (*%* score 20 – 28) | 2.4 | 17.4 | 8.8 | 3.7 | 0.8 |  |  |  |
| Severe (*%* score 29 – 63) | 1.6 | 6.5 | 10.5 | 4.3 | 0.0 |  |  |  |

*Note.* ADHD = Attention Deficit Hyperactivity Disorder; DSM-5 = Diagnostic and Statistical Manual of Mental Disorders, Fifth Edition.

Symptoms of ADHD are measured with the Attention Deficit Hyperactivity Disorder rating scale (ARS). Neuroticism, extraversion, openness, agreeableness and conscientiousness are measured with the Neuroticism-Extraversion-Openness Five Factor Inventory (NEO-FFI). Symptoms of depression are measured with the Beck Depression Inventory II (BDI-II-NL).

^1^ DSM-5 ADHD classification in adulthood when **≥** 5 symptoms of inattention and/or **≥** 5 symptoms of hyperactivity and impulsivity were classified as being present in the last six months.

^2^ DSM-5 ADHD classification in childhood when **≥** 6 symptoms of inattention and/or **≥** 6 symptoms of hyperactivity and impulsivity were classified as being present when 0 – 12 years old.

** p* < .05. Post-hoc Bonferroni significant group differences: ^a^ *versus* No ADHD group, ^b^ *versus* Subthreshold ADHD group and ^c^ *versus* Adult-only ADHD group.
