# Supplementary material for: Financial decision-making in a community sample of adults with and without current symptoms of ADHD
Source: PLoS One. 2020 Oct 12;15(10):e0239343. doi: 10.1371/journal.pone.0239343 (PMC7549773; doi:10.1371/journal.pone.0239343)
Supplement: S2 Table — (DOCX) [file pone.0239343.s002.docx]

**S2 Table. Pearson correlations in the total sample**

|  | 1. | | 2. | | 3. | | 4. | | 5. | | 6. | | 7. | | 8. | | 9. | | 10. | |
| --- | --- | --- | --- | --- | --- | --- | --- | --- | --- | --- | --- | --- | --- | --- | --- | --- | --- | --- | --- | --- |
| 1. Symptoms of ADHD | - | |  | |  | |  | |  | |  | |  | |  | |  | |  | |
| 2. Age | -.329 | ** | - | |  | |  | |  | |  | |  | |  | |  | |  | |
| 3. Sex^1^ | -.034 |  | -.040 |  | - | |  | |  | |  | |  | |  | |  | |  | |
| 4. Education | .087 | * | -.254 | ** | .000 |  | - | |  | |  | |  | |  | |  | |  | |
| 5. Income | -.109 | ** | .248 | ** | -.348 | ** | .213 | ** | - | |  | |  | |  | |  | |  | |
| 6. Neuroticism | .401 | ** | -.241 | ** | .209 | ** | -.055 | * | -.324 | ** | - | |  | |  | |  | |  | |
| 7. Extraversion | -.032 |  | -.142 | ** | -.003 |  | .084 | * | .090 | * | -.417 | ** | - | |  | |  | |  | |
| 8. Openness | .058 | * | -.070 | * | .083 | * | .352 | ** | .072 | * | -.030 |  | .114 | ** | - | |  | |  | |
| 9. Agreeableness | -.304 | ** | .137 | ** | .328 | ** | .042 |  | -.032 |  | -.238 | ** | .271 | ** | .124 | ** | - | |  | |
| 10. Conscientiousness | -.341 | ** | .183 | ** | .087 | * | .046 |  | .236 | ** | -.369 | ** | .231 | ** | -.009 |  | .289 | ** | - | |
| 11. Depression | .339 | ** | .058 | * | .136 | ** | -.082 | * | -.141 | ** | .531 | ** | -.351 | ** | -.010 |  | -.136 | ** | -.192 | ** |

*Note.* ADHD = Attention Deficit Hyperactivity Disorder. Symptoms of ADHD are measured with the Attention Deficit Hyperactivity Disorder rating scale (ARS) current version. Neuroticism, extraversion, openness, agreeableness and conscientiousness are measured with the Neuroticism-Extraversion-Openness Five Factor Inventory (NEO-FFI). Symptoms of depression are measured with the Beck Depression Inventory II (BDI-II-NL).

^1^ Sex: 0 = male, 1 = female

** p* < .05, ** *p* < .001
